# The role of susceptibility-weighted imaging & contrast-enhanced MRI in the diagnosis of primary CNS vasculitis: a large case series

**DOI:** 10.1038/s41598-024-55222-2

**Published:** 2024-02-27

**Authors:** Sushant Agarwal, Leve Joseph Devarajan Sebastian, Shailesh Gaikwad, M. V. Padma Srivastava, M. C. Sharma, Manmohan Singh, Rohit Bhatia, Ayush Agarwal, Jyoti Sharma, Deepa Dash, Vinay Goyal, Achal K. Srivastava, Manjari Tripathi, Vaishali Suri, Mamta B. Singh, Chitra Sarkar, Ashish Suri, Rajesh K. Singh, Deepti Vibha, Awadh K. Pandit, Roopa Rajan, Anu Gupta, A. Elavarasi, Divya M. Radhakrishnan, Animesh Das, Vivek Tandon, Ramesh Doddamani, Ashish Upadhyay, Venugopalan Y. Vishnu, Ajay Garg

**Affiliations:** 1 Department of Neuroimaging and Interventional Neuroradiology, All India Institute of Medical Sciences, New Delhi, India; 2 Department of Neurology, All India Institute of Medical Sciences, New Delhi, India; 3 Department of Pathology, All India Institute of Medical Sciences, New Delhi, India; 4 Department of Neurosurgery, All India Institute of Medical Sciences, New Delhi, India; 5 Department of Biostatistics, All India Institute of Medical Sciences, New Delhi, India

**Keywords:** Vasculitis, PCNSV, DSA, PCNSV, Venulitis, SWI, Microhemorrhages, Gadolinium, Diseases of the nervous system, Neurology

## Abstract

Primary CNS Vasculitis (PCNSV) is a rare, diverse, and polymorphic CNS blood vessel inflammatory condition. Due to its rarity, clinical variability, heterogeneous imaging results, and lack of definitive laboratory markers, PCNSV diagnosis is challenging. This retrospective cohort analysis identified patients with histological diagnosis of PCNSV. Demographic data, clinical presentation, neuroimaging studies, and histopathologic findings were recorded. We enrolled 56 patients with a positive biopsy of CNS vasculitis. Most patients had cerebral hemisphere or brainstem symptoms. Most brain MRI lesions were bilateral, diffuse discrete to confluent white matter lesions. Frontal lobe lesions predominated, followed by inferior cerebellar lesions. Susceptibility-weighted imaging (SWI) hemorrhages in 96.4% (54/56) of patients, either solitary microhemorrhages or a combination of micro and macrohemorrhages. Contrast-enhanced T1-WIs revealed parenchymal enhancement in 96.3% (52/54 patients). The most prevalent pattern of enhancement observed was dot-linear (87%), followed by nodular (61.1%), perivascular (25.9%), and patchy (16.7%). Venulitis was found in 19 of 20 individuals in cerebral DSA. Hemorrhages in SWI and dot-linear enhancement pattern should be incorporated as MINOR diagnostic criteria to diagnose PCNSV accurately within an appropriate clinical context. Microhemorrhages in SWI and venulitis in DSA, should be regarded as a potential marker for PCNSV.

## Introduction

Primary central nervous system vasculitis (PCNSV) is a rare, heterogeneous, and polymorphic inflammatory disorder of unexplained etiology exclusively affecting the blood vessels of the brain and the spinal cord with no other systemic involvement^1–3^. The clinical presentation varies widely, and the imaging results are diverse, making the diagnosis challenging. Typically, patients manifest a combination of symptoms such as headaches, seizures, and focal neurological deficits, and there is often a substantial delay between the onset of symptoms and the eventual diagnosis of PCNSV^4–6^. Diagnosis of PCNSV may require invasive procedures like DSA and brain biopsy.

No single investigation, whether biochemical, serological, immunological, or imaging-based, can definitively confirm PCNSV. A meningo-cortical biopsy remains the gold standard for establishing the diagnosis^5^. However, brain biopsy and the relatively inaccessible spinal cord biopsy are invasive, potentially risky, and may not always be feasible.

There is currently no universally accepted or thoroughly validated set of clinical diagnostic criteria for PCNSV^7,8^. While there is no consensus on criteria that definitively define the disease, numerous studies and published case reports have employed diagnostic criteria based on imaging techniques such as CTA, MRA, or DSA, without resorting to biopsy^4,5,8–14^. Some studies suggest relying on HPE^7,14–16^. The variation in criteria for patient inclusion and the absence of randomized controlled trials in this patient population make it challenging to interpret and optimize treatment approaches.

Our research group had previously published clinical findings and treatment outcomes in individuals with PCNSV, with confirmation achieved through either brain biopsy or cerebral digital subtraction angiography^17^. In this manuscript, we have described the imaging findings in 56 patients with confirmed PCNSV via biopsy. Furthermore, we propose practical clinico-radiological criteria for diagnosing PCNSV.

## Material and methods

### Patient recruitment

A retrospective study was conducted by searching the neuroradiology Picture Archiving and Communication System (PACS), AIIMS PCNSV registry, and neuropathological database for the term 'cerebral vasculitis'. Patients in whom PCNSV was diagnosed histopathologically between January 1, 2010, and December 31, 2021, were identified. Subsequently, the medical records of these patients were carefully reviewed, excluding those with an alternative diagnosis or not meeting the diagnostic criteria for PCNSV. This cohort was then independently reviewed by two experienced neurologists (VY, AA), and patients were included in the study only if there was a consensus regarding the diagnosis of PCNSV. Finally, the clinical course was considered, and only patients with progressive disease or frequent relapse lasting more than 12 weeks were confirmed to have PCNSV, thereby ruling out RCVS. The Institute Ethics Committee (IEC) granted approval for this study and also waived the need for informed consent for this retrospective analysis.

The study enrolled individuals who met the following inclusion criteria: (1) adult patients aged 18 years or older, who had a recent history or current presence of an unexplained neurological deficit; (2) individuals with evidence of vasculitis in a central nervous system biopsy specimen; and (3) exclusion of secondary vasculitis based on biochemical investigations. Participants who showed evidence of systemic vasculitis or other conditions that mimic primary central nervous system vasculitis, those with a hypercoagulable state, or those whose clinical, neuroimaging, or biopsy findings could be attributed to infections or other causes were excluded from the study.

Demographic information, clinical presentation, initial CSF findings, neuroimaging studies including MRI and DSA, as well as histopathological findings from cerebral brain biopsy samples were gathered. Differential diagnoses were ruled out through CSF analysis. The age of disease onset was defined as the age at which the initial clinical manifestations became evident. All methods were performed in accordance with the IEC guidelines and regulations.

### Blood investigations

Serum vasculitis profile (ANA, lupus anticoagulant, anti-cardiolipin antibody, anti-ds DNA and ANCA) was done in all patients to exclude a systemic vasculitis process. Furthermore, we conducted investigations to exclude common chronic infectious and inflammatory causes, including HIV, hepatitis B and C serology, and syphilis (VDRL).

Additional serological tests such as HLA-B51, RA factor, anti-SSA, anti-SSB, and cryoglobulins were performed based on the specific clinical characteristics and profile of each patient. Serum NMO and MOG antibodies were tested in all patients with spinal cord manifestations.

### CSF

CSF examination was considered abnormal when the cell count exceeded five cells per microliter (µL), and protein content was higher than 45 mg per deciliter (mg/dL).

### Imaging evaluation

The imaging investigations (MRI and IADSA) were reviewed and analyzed by two neuroradiologists (SA, AG). MRIs were reviewed for distribution and patterns of white matter changes, hemorrhages, and enhancing lesions in the different brain and spinal cord regions, and the data were captured on a predefined structured system.

*Cerebral microhemorrhages* were identified as small, round foci of hypointense signals on GRE/SWI, measuring up to 10 mm, located within the cerebral parenchyma in either deep or lobar regions^18^. Larger hemorrhagic lesions observed on GRE/SWI were classified as macrohemorrhages, and their presence or absence was documented in different brain regions.

*Acute SAH* was interpreted as a FLAIR hyperintense signal within one or more brain sulci. When a curvilinear rim of hypointensity is observed in the subarachnoid space on GRE/SWI imaging, it is classified as subacute SAH^19^.

*Punctate enhancements were* defined as multiple small, uniform, dot-like enhancements with a diameter of less than 3 mm, while round enhancements ranging from 3 to 9 mm were classified as nodular enhancements^20^. *Perivascular space (PVS) enhancement was indicated by the linear enhancement along the pathways of perforating vessels.*

The *Ischemic lesion* pattern was categorized according to a rating scale adopted from Szabo et al.^21^, which included the following classifications: (1) a large lesion affecting the cortex, (2) a subcortical lesion, either with or without additional smaller lesions, (3) a large lesion involving the cortex with additional smaller lesions, (4) disseminated lesions in distal cortical regions, and (5) multiple lesions in areas with a hemodynamic risk.

The *DSAs* were carefully evaluated, and the diseased vessels were categorized according to the territory involved, proximal vs. distal vessel involvement, unilateral vs. bilateral involvement, anterior vs. posterior circulation involved, and arterial or venous phase abnormality.

Positive DSA results were determined by abnormalities in either the arterial or venous phase. Arterial phase findings included isolated or multiple steno-occlusive changes, irregularities, a beaded or pearlescent appearance and aneurysms. Venous phase abnormalities were dilatation and tortuosity of small veins, puddling and staining of contrast, and prominence of transmedullary veins with irregular outlines, referred to as parenchymal venous phase abnormalities or pseudophlebitis. Additionally, the findings from DSA were compared and correlated with those from SWI on MRI.

Each patient's MRI study was also compared to their initial MRI and subsequent MRIs to detect any changes in lesions, hemorrhages, or patterns of enhancement.

### Histopathology

Biopsies were categorized into two types: targeted biopsies, which were obtained from regions of the brain showing abnormalities on imaging, and blinded biopsies, which were taken from non-dominant frontal or temporal pole areas. A biopsy was considered positive if transmural inflammation in small or medium-sized blood vessels within the meningocortical region was present, and the neuropathologist’s comprehensive assessment confirmed the diagnosis of PCNSV. The different HPE patterns of inflammation were recorded as previously described and included granulomatous patterns with/without amyloid deposition, lymphocytic patterns and necrotizing vasculitic changes.

### Statistical analysis

Categorical data were presented as percentages, while continuous data were expressed as median values along with the interquartile range. When applicable, we utilized chi-square or Fisher's exact tests to examine differences in categorical variables, and the Mann–Whitney test was employed to analyze continuous variables. A p-value less than 0.05 was deemed statistically significant. All statistical analyses were performed using SPSS version 28.0.

## Results

A total of 302 individuals with suspected PCNSV underwent screening, identifying 56 cases that met the inclusion and exclusion criteria. The demographic and clinical characteristics are detailed in Table 1. The median age at presentation was 34 years, with a male predominance (4.6:1). The median time from the onset of the initial symptom to diagnosis was 18 months. The most frequently reported presenting symptom was seizures (69.6%), followed by headaches (62.5%). The majority of our patients experienced progressive deficits (91.1%).

**Table 1:** Demographics & clinical characteristics.

	PCNSV cohort (n = 56)
Sex (Male: Female)	46:10 (4.6:1)
Age, median (range) IQR in years	34 (18–69)
	27–44 years
Age of Onset, median (range) IQR in years	28.3 (22.9–38.2)
	22.58–38.66 years
Age of Diagnosis, median (range) IQR in years	32.5 (9–68.9)
	25–40 years
Interval b/w symptom onset & diagnosis, median (range) in months, IQR	18 (0.2–168)
	6–43 months
Presenting symptoms [number(%)]	
Seizure	39 (69.6%)
Headache	35 (62.5%)
Hemiparesis	28 (50.0%)
Cognitive impairment	20 (35.7%)
Paraparesis	9 (16.1%)
Ataxia	11 (19.6%)
Facial nerve palsy	10 (17.9%)
Visual symptoms	8 (14.3%)
Clinical course	
Static	5 (8.9%)
Progressive	51 (91.1%)
Clinical episodes	
Monophasic	8 (14.3%)
Multiphasic	48 (85.7%)

Cerebrospinal fluid (CSF) examination was available in 31 out of 56 patients, with abnormal findings observed in 26 (83.9%). These abnormalities included pleocytosis in 12 cases, elevated protein levels in 25 cases (median value: 82.5 mg/dl, ranging from 29 mg/dl to 444 mg/dl) and normal glucose in all patients. Gram staining, TB-PCR, VDRL, Cryptococcal antigen testing, and cultures yielded negative results in all patients (see Table 2).

**Table 2:** Investigations.

CSF examination	31 (56.4%)
Abnormal	26/31 (83.9%)
Abnormal cells	13 (50%)
Abnormal protein	26 (100%)
DSA Done	20/56 (35.7%)
Abnormal	19/20 (95%)
Unilateral involvement	1 (5.3%)
Bilateral involvement	18 (94.7%)
Arterial irregularities	0
Beaded appearance-	0
Vessel occlusion	0
Venous phase abnormality	19 (95%)
Biopsy	
Granulomatous	24 (42.9%)
Lymphocytic	31 (55.4%)
Necrotizing-	1 (1.8%)

### Imaging

The initial site of CNS involvement was primarily the brain in 83.9% of cases, the spinal cord in 8.9%, and both the brain and spinal cord simultaneously in 7.1% of cases (Table 3). The most common lesion distribution was bilateral and diffuse, followed by a predominantly unilateral, without any significant difference between the distribution of lesions. Combined supratentorial and infratentorial involvement was the most common finding on MRI. Involvement of both supratentorial and infratentorial regions was the most common finding observed on MRI, with only two cases displaying isolated infratentorial involvement.

**Table 3:** MRI in PCNSV.

	All cases, 56
MRI Done	56 (100%)
Median number of MRI studies (IQR)	3 (2–6)
First CNS region of involvement	
Brain	47 (83.9%)
Spinal cord	05 (8.9%)
Both brain & spinal cord	4 (7.1%)
Distribution	
Focal bilateral	7 (4.7%)
Diffuse bilateral	98 (65.3%)
Predo. Unilateral	34 (22.7%)
Unilateral	11 (7.3%)
Involvement	
Supratentorial only	12 (21.4%)
Infratentorial only	2 (3.6%)
Both	42 (75%)
Parenchymal hemorrhages	**53 (96.4%)**
Microhemorrhages	23 (41.1%)
Both Micro- & Macro hmgs	31 (55.4%)
None	2 (3.6%)
ICH	18 (32.1%)
SAH	0
Diffusion restriction	11 (19.6%)
Infarct pattern	
Cortical	2 (3.6%)
Subcortical	32 (57.1%)
Cortical & Subcortical	22 (39.3%)
GM involvement	
Cortical GM	12 (21.4%)
Deep GM	23 (41.1%)
Both	19 (33.9%)
None	2 (3.6%)

**Figure 1:**
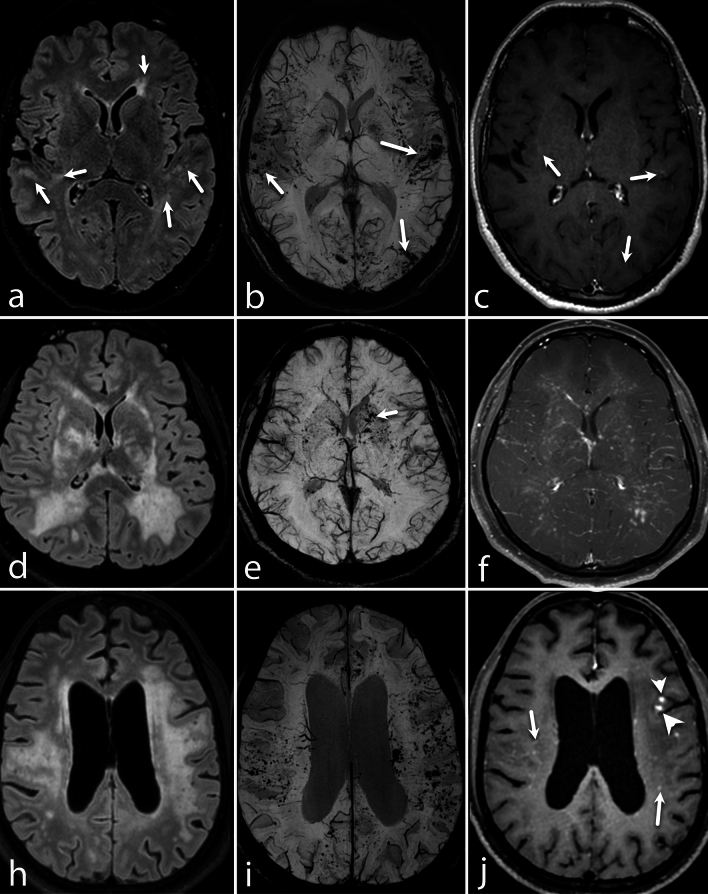
Different patterns of FLAIR hyperintensities, SWI hemorrhages, and enhancements in PCNSV. (**a**–**c**) A 28-year-old man presented with headaches for the past 3.5 years. The axial FLAIR (**a**) image shows multi-focal subtle areas of FLAIR hyperintensities in subcortical and periventricular white matter (arrows) of bilateral cerebral hemispheres. The axial SWI image (**b**) shows multiple microhemorrhages (arrows) in bilateral cerebral hemispheres. Axial post-gadolinium T1-WI (**c**) shows punctate foci of enhancement (arrows) in bilateral cerebral hemispheres. (**d**–**f**) A 23-year-old man presented with acute onset vision loss in the left eye. The axial FLAIR image (**d**) shows confluent symmetrical pattern of white matter hyperintensity in the periventricular white matter of the bilateral fronto-parietal lobe, basal ganglia, internal capsule, and external capsule. The axial SWI (**e**) shows multiple foci of microhemorrhages (arrow) predominantly in bilateral basal ganglia. Post-contrast image (**f**) shows a diffuse perivascular and nodular enhancement pattern in the bilateral cerebral hemisphere. (**g**–**i**) A 37-year-old man presented with behavioral changes for the past 6 months. The axial FLAIR (g) image shows large confluent and discrete areas of periventricular and subcortical white matter hyperintensity in bilateral frontoparietal lobes. The axial SWI (**b**) shows diffuse punctate foci of microhemorrhages in the bilateral cerebral hemisphere. On the post-contrast image (**c**), a few nodular (arrowheads) and punctate foci (arrows) of enhancement are seen.

**Figure 2:**
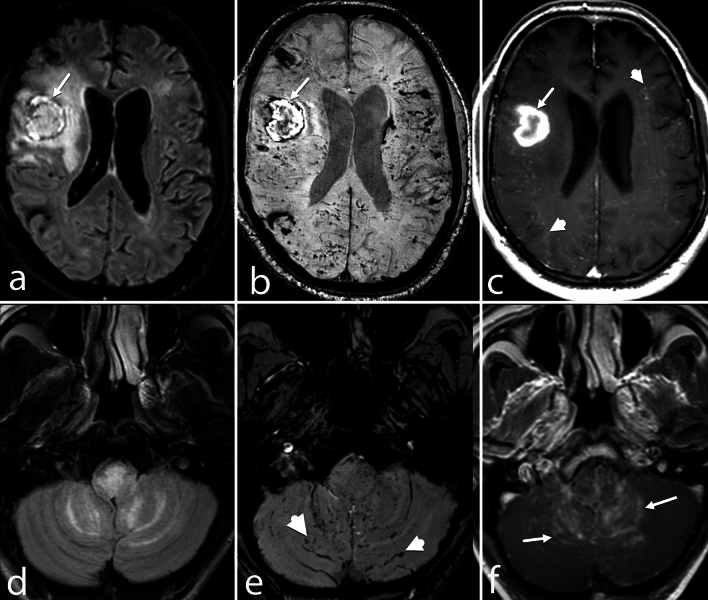
Different patterns of FLAIR hyperintensities, SWI hemorrhages, and enhancements in PCNSV. (**a**–**c**) A 48-year-old man presented with recurrent episodes of neurological deficits. The axial FLAIR image (**a**) shows a well-defined heterogeneously hyperintense lesion (arrow) in the right frontal lobe with adjacent edema. A few discrete white matter hyperintensities are also seen in the left frontal and right occipital lobes. On SWI image (**b**), a macrohemorrhage is seen in the right cerebral hemisphere (arrow) with punctate and linear foci of microhemorrhages in bilateral cerebral hemispheres. Post-contrast image (c) shows diffuse dot-linear enhancement (arrrowheads) in the bilateral cerebral hemisphere along with subacute hematoma in the right frontal lobe (arrow). (**d**–**f**) A 38-year-old man presented with headache, vomiting, hiccups, and ataxia for the past 3.5 months. MRI FLAIR axial image (**a**) shows FLAIR hyperintensity in the medulla and along the folia of the bilateral cerebellar hemisphere. SWI image (**b**) shows dot-linear blooming foci (arrowheads) along the folia of the cerebellum with few punctate blooming in the medulla. Post-contrast study (**c**) shows diffuse leptomeningeal enhancement (arrows) in the cerebellum with punctate foci of enhancement in the medulla and cerebellum.

**Figure 3:**
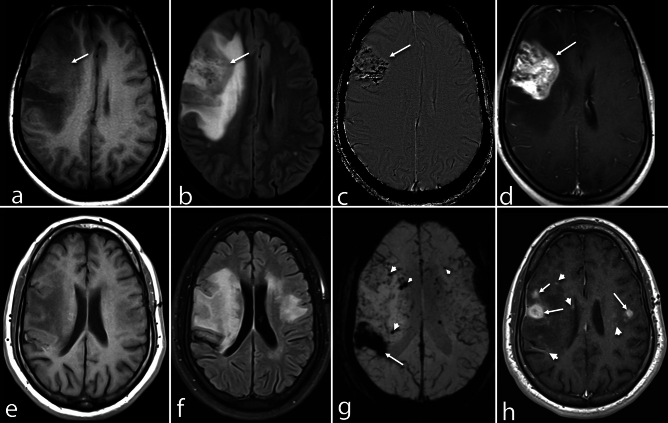
Atypical PCNSV patterns. (**a**–**d**) A 28-year-old woman presented with sudden onset left hemiparesis. Axial T1-WI (**a**) and FLAIR image (**b**) show a poorly defined heterogeneously mass lesion (arrows), isointense in T1-WI and hyperintense in FLAIR (**b**) in the right frontal lobe with adjacent edema causing mass effect on right lateral ventricles. Axial SWI phase image (**c**) shows multiple foci of microhemorrhages (arrow) in the lesion. In post-contrast study (**d**), the lesion shows intense heterogenous enhancement (arrow). (**e**–**h**) An 18-year-old boy with a history of progressive cognitive decline and headaches presented with sudden onset left hemiparesis. The axial T1-WI (**e**) and FLAIR image (**f**) show well-defined signal abnormalities, hypointense on T1-WI and hyperintense in FLAIR image, in the bilateral frontoparietal lobes' subcortical and periventricular white matter. The axial SWI image (**g**) shows macrohemorrhages (arrowheads) in the right parietal lobe with foci of microhemorrhages (arrows) in bilateral cerebral hemispheres. Axial post-contrast image (**h**) shows the nodular (arrowheads) and dot-linear (arrows) patterns of enhancement.

Microhemorrhages and/or macrohemorrhages were detected in 53 out of 55 patients (96.4%). The combined presence of micro and macrohemorrhages was the most common pattern, followed by isolated microhemorrhages (Figs. 1, 2, 3). No cases presented with exclusively macrohemorrhages, and SAH was not observed in any patient.

Regarding infarct patterns, subcortical white matter involvement was the most prevalent, followed by combined cortical and subcortical involvement. Among the patients with available diffusion-weighted MRI scans (54 out of 56), 11 cases (20%) exhibited foci of restricted diffusion.

At the time of initial presentation, contrast-enhanced MRI examinations were conducted in 54 out of 56 patients. Parenchymal enhancement was evident in 52 of these patients. The most common enhancement pattern was dot-linear (87%), followed by nodular (61.1%), perivascular (24.5%), and patchy enhancement (16.7%). Leptomeningeal and pachymeningeal enhancements were observed in 20.8% and 7.6% of patients, respectively (as documented in Table 4). No correlations were identified between SWI blooming, enhancement patterns, or FLAIR signal abnormalities.

**Table 4:** Enhancement pattern in brain lesions.

	Definite vasculitis
Contrast-enhanced scan available	54/56 (96.4%)
Parenchymal enhancement	52 (96.3%)
Dots-linear	47 (87%)
Nodular	33 (61.1%)
Patchy	9 (16.7%)
Tumefactive	3 (5.6%)
Perivascular enhancement	14 (25.9%)
Subependymal Enhancement	6 (11.1%)
Leptomeningeal enhancement	11 (20.8%)
Focal	9 (16.7%)
Diffuse	2 (3.7%)
Pachymeningeal enhancement	4 (7.6%)
Focal	3 (5.6%)
Diffuse	1 (1.9%)

### Spinal cord MRI

**Figure 4:**
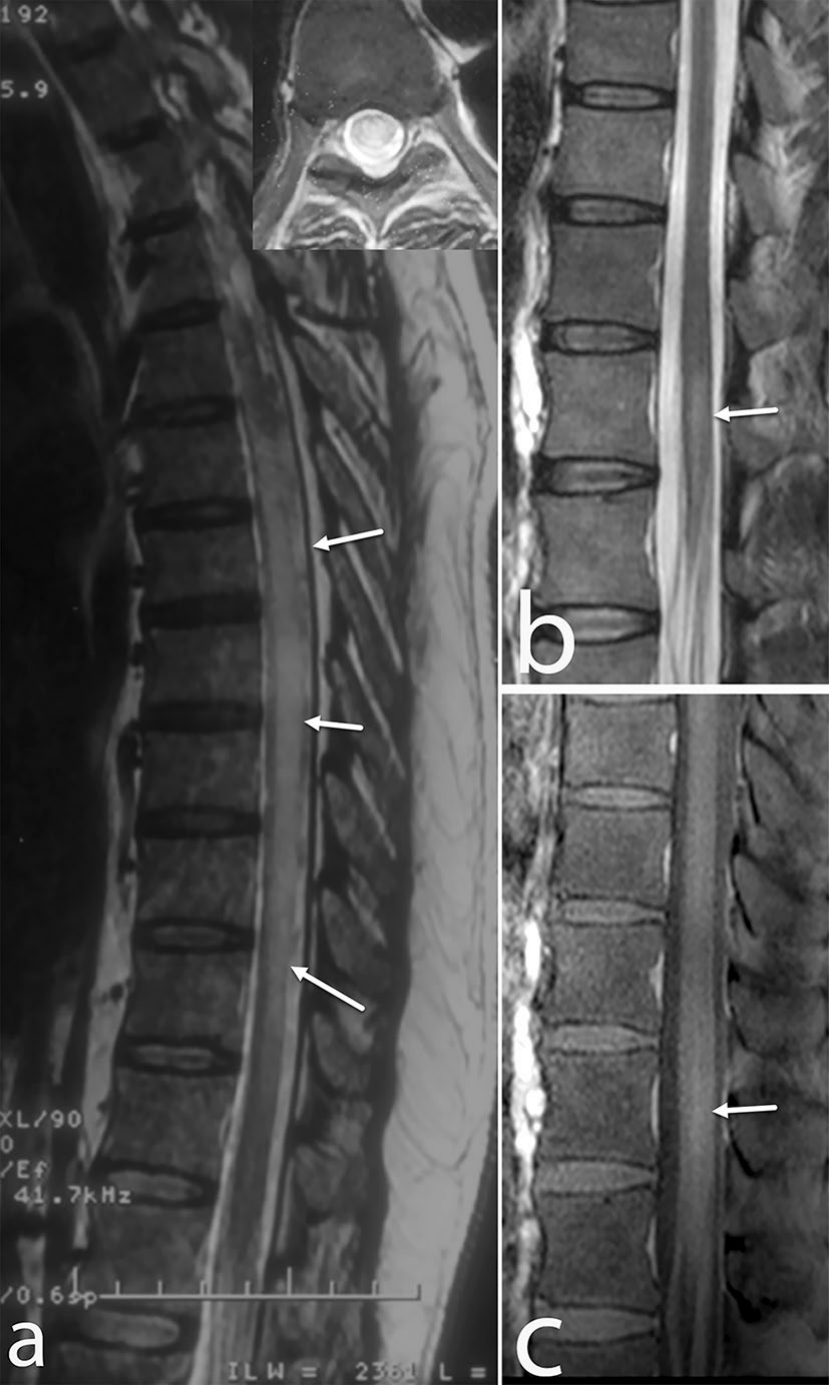
Spinal cord involvement in PCNSV cases. Sagittal T2-WI (**a**) shows long segment cord hyperintensity and expansion (arrows) in the upper and mid-dorsal cord. In another patient of cerebral PCNSV, sagittal T2-WI of the lower dorsal cord (**b**) shows short segment cord hyperintensity (arrow), which enhances mildly (arrow) following gadolinium administration (**c**).

Among the 56 patients, spinal cord MRIs were performed in 33 individuals. Of these, 21 were conducted during the initial presentation, and 12 were carried out at various time points during follow-up. Among the 21 spinal MRIs performed at the initial presentation, 11 displayed abnormalities (9 symptomatic cases and 2 asymptomatic cases). In contrast, of the 12 MRIs performed during follow-up, only one exhibited abnormality, and this case was symptomatic. The dorsal spinal cord (8 out of 12) and conus region (7 out of 12) were the most commonly affected areas, followed by the cervical cord (3 out of 12). The predominant lesion pattern observed was a single short segment (6 out of 12), followed by multifocal lesions (4 out of 12) and single long-segment lesions (2 out of 12) (Fig. 4). Additionally, spinal cord expansion and contrast enhancement were evident in 7 of the 12 patients for each characteristic [Supplementary Table 2].

### Cerebral DSA

**Figure 5:**
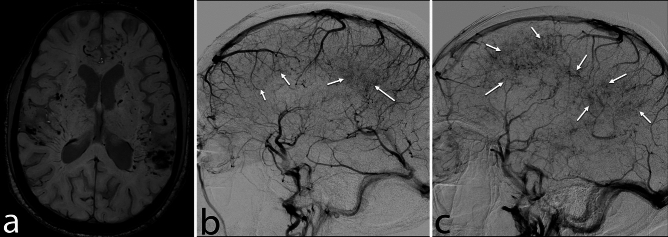
Bilateral venulitis. A 33-year-old man presented with recurrent episodes of neurological deficits with chronic headaches for the past 6 months. Axial SWI image (**a**) shows multiple foci of microhemorrhages in bilateral cerebral hemispheres (predominantly in subcortical location). DSA images (**b**, **c**) show puddlings of contrast in the delayed venous phase of angiogram (arrowheads) in bilateral cerebral hemispheres (pseudophlebitis).

**Figure 6:**
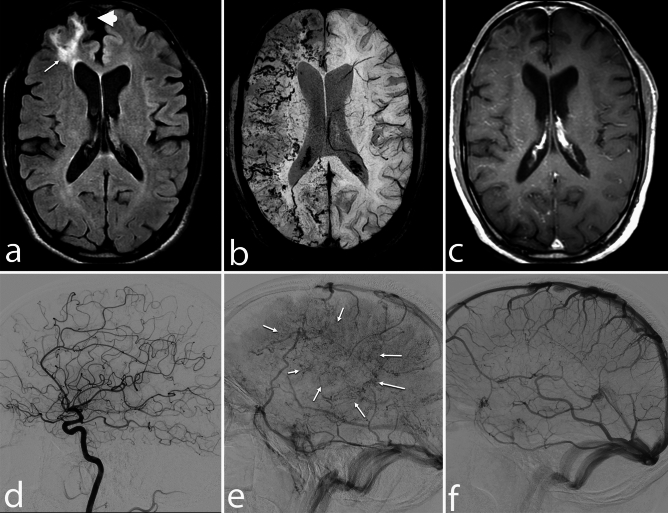
Atypical unilateral PCNSV. A 39-year-old man presented with chronic headaches and sudden onset left hemiparesis. In MRI, axial FLAIR image (**a**) shows a well-defined area of hyperintensity in the subcortical and periventricular white matter of the right anterior frontal lobe (arrow) with dilation of adjacent cortical sulci (arrowhead) and iplitaeral frontal horn. Axial SWI image (**b**) shows multiple curvilinear and punctate foci of blooming in the right cerebral hemisphere. Note that the extent of blooming on SWI is quite extensive compared to FLAIR signal abnormality. Post-contrast T1-WI (**c**) shows a perivascular and punctate pattern of enhancement in the right cerebral hemisphere. Cerebral DSA images (**d**, **e**) of the right cerebral hemisphere show a pseudophlebitis pattern in delayed venous phase (**e**) with no obvious abnormality in the arterial phase (**d**). DSA image (**f**) shows a normal venous angiogram of the left cerebral hemisphere. These findings are compatible with the unilateral pattern of PCNSV.

Cerebral DSA was conducted in 20 of the 56 patients, revealing abnormalities in 19 (95%). All 19 patients with abnormal DSA exhibited parenchymal venous phase abnormalities, with bilateral abnormalities evident in 18 cases (Fig. 5) and unilateral abnormalities in one case (Fig. 6). Notably, the posterior circulation was relatively spared, and most patients displayed vascular abnormalities in supratentorial regions [Table 2].

### Histopathology

Regarding histopathology, brain biopsies were performed on 80 patients, with 69 of them being targeted and 11 being blind biopsies taken from frontal or temporal poles. Among these, 56 patients (70%) showed positive results for PCNSV, while the remaining biopsies did not reveal any evidence of vasculitis or other brain pathology. Although the targeted biopsy had a higher positivity rate than the blind biopsy (73.9% vs. 45.4%), this difference did not reach statistical significance. The most common subtype of PCNSV observed was lymphocytic vasculitis (55.4%), followed by granulomatous vasculitis (42.9%) and necrotizing vasculitis (1.8%) (Table 2).

## Discussion

This is the largest cohort of 56 patients diagnosed with PCNSV through biopsy in India. None of the enrolled patients had clinical manifestations or serum biomarkers indicative of systemic involvement, effectively excluding the possibility of secondary vasculitis.

The clinical manifestations in our series shared similarities with previous studies, including gender distribution and a diverse range of clinical symptoms. Our study's median age of onset was younger (28.66 years) than previous studies^4,5,22^. Seizures were the most common clinical manifestation, followed by headaches and persistent motor deficits (hemiparesis).

The mainstay of imaging of CNS vasculitis is MRI and DSA. MRI findings in PCNSV are exceptionally diverse and frequently include multiple lesions, which are usually bilateral, asymmetrical, and predominantly in the supratentorial compartment involving both GM and WM, particularly the subcortical and deep WM^5,23^. Additionally, multiple micro-and macro-hemorrhages may be present, best detected by SWI. Less commonly, PCNSV may manifest as a primary intraparenchymal hemorrhage, SAH, or single or multiple parenchymal masses. Perfusion imaging may detect areas of hypoperfusion in suspected cases of vasculitis, even in the absence of parenchyma changes on conventional MR imaging^2^. Vessel wall imaging, utilizing 3D or 2D thin post-contrast T1-WI with fat saturation and flow compensation, may reveal vessel wall thickening and enhancement^24^ and may assess vasculitis activity.

While DSA is regarded as the gold standard for imaging assessment in PCNSV, it has limitations in terms of sensitivity and specificity, both typically ranging around 25% to 35%^25–29^. The classic DSA appearance in CNS vasculopathy includes segmental or multiple focal stenoses of vessels, with or without intermittent dilated segments that create a "string of pearls" appearance. Other findings that may be observed in CNS vasculitis encompass vessel wall irregularities, occlusion of small arteries or non-perfused areas, narrowing of multiple vessels in single tributary regions, collateral blood flow, and delayed circulation time in specifically affected regions^14,23,30,31^. However, the sensitivity of DSA is poor, particularly when the affected vessel diameter is less than 500 microns^32^. In fact, up to 40% of cases with biopsy-confirmed vasculitis may show entirely normal DSA results^2,5,8,23,25,29,33–39^. Changes may only become evident in a subsequent DSA for a few patients. CT-based or MR angiography, although less studied with histopathological confirmation, appears to be even less sensitive^40–43^.

In the present study, MRI was abnormal in all cases. The brain was the first region of CNS involvement in most patients. In the brain MRI, the most common distribution of lesions was bilateral, characterized by diffuse discrete to confluent white matter lesions occurring in both the supratentorial and infratentorial compartments. Lesions in the frontal lobe were most common, followed by involvement of the inferior cerebellar region. In a systematic review of published cases of PCNSV, cerebral MRI was found to be abnormal in 93% of patients, indicating that it can occasionally appear unremarkable (a normal MRI result)^44^). Additionally, CSF abnormalities were detected in 74% of cases^26^.

Depending on the nature and size of the affected blood vessels (i.e., arteries or veins), MRI could reveal various patterns or combinations, such as infarct of entire vessel territory, involvement of watershed regions, lacunar infarcts, tumor-like lesions, intracerebral hemorrhage (ICH), cerebral microhemorrhages, venous thrombosis, and lesions within the spinal cord. Furthermore, after administering contrast agents, MRI scans might show pachymeningeal, leptomeningeal, gyral, patchy, nodular, punctate, or curvilinear enhancements.

In the present study, SWI revealed the presence of parenchymal hemorrhages in nearly all cases of PCNSV, which is significantly higher compared to findings in existing literature. In the French cohort, the reported incidence of hemorrhages on MRI ranged from 10.8% to 63.6%^19^. Schuster et al. reported that among 31 patients, four had parenchymal hemorrhages (three microhemorrhages and one macrohemorrhage), and one patient had sulcal SAH^45^. A study on PCNSV found that 12.2% of patients had intracerebral hemorrhages (ICH) at or near the time of presentation^46^, while another study reported a much higher incidence of 63.6%^19^. Notably, ICH was more frequently observed in women, and necrotizing vasculitis was the predominant histopathological pattern associated with ICH^46^. These findings suggest that the incidence of ICH in PCNSV might be underestimated, and the inclusion of an SWI scan should be considered when PCNSV is suspected. The increased incidence of hemorrhages in our study can partly be attributed to the utilization of SWI, which is five times more sensitive than T2* imaging for detecting hemorrhages, or it may be due to underestimation in other studies. Another possible explanation could be selection bias, as patients with hemorrhages might have been more likely to undergo DSA or brain biopsy in our setting; other etiologies were considered when there were no microhemorrhages.

SWI is highly sensitive to detect microhemorrhages, a manifestation of small vessel cerebral disease (SVD) commonly associated with conditions like hypertensive arteriopathy (arteriolosclerosis) and cerebral amyloid angiopathy. Certain microhemorrhages patterns, such as linear/lace-like blooming on SWI with asymmetrical margins and perilesional FLAIR signal, as well as linear, lace-like, or central dot-like enhancement at sites of SWI blooming, have been suggested to support a diagnosis of CNS vasculitis^47^. However, our study did not find a correlation between dot-linear enhancement and areas of blooming on SWI. We posit that the susceptibility signals in SWI primarily represent hemorrhages rather than free radicals from leukocyte infiltrates. Interestingly, even after treatment with steroids, the white matter hyperintensities may resolve, but microbleeds detected on SWI imaging tend to persist^48^.

Parenchymal enhancement was observed in 54 out of 56 cases in which contrast-enhanced T1-WIs were available. The miliary pattern of nodular enhancement along the perivascular VR spaces is also described in PCNSV^19^. Terada et al.^49^ and Sostak et al.^50^ have also reported a similar miliary pattern of cerebral enhancement along the VR spaces, probably due to disruption of small-sized vessels implicating direct endothelial cell injury or by angiocentric infiltration by histiocyte and B & T lymphocytes.

In the present study, leptomeningeal enhancement was observed in 20.8% of patients, and none exhibited abnormalities in the arterial phase of DSA. This incidence of leptomeningeal enhancement is more than reported in the existing literature. Leptomeningeal enhancement is believed to be associated with widespread inflammation and vasculitic changes within the leptomeninges. This particular enhancement pattern has been identified in PCNSV patients as in case reports^31,51–53^ or case series^54^. For instance, Salvarani et al. reported strong leptomeningeal enhancement in 8 out of 101 patients with PCNSV^54^. Notably, these patients were more likely to have a rapid onset of symptoms, a prompt response to therapy, and achieve better clinical outcomes. None of these 8 cases had abnormalities in DSA or MRA, or both, suggesting that leptomeningeal enhancement may potentially signify small vessel vasculitis, which falls beyond the resolution of DSA. In the present study, pachymeningeal enhancement was observed in 7.6% of patients. Pachymeningeal enhancement refers to the enhancement of the dura-arachnoid layer, which occurs due to inflammation in the blood vessels supplying the meninges. This phenomenon has been observed in various pathological conditions, such as infiltration by tumor cells or inflammatory cells.

In the present study, spinal cord MRIs were available in 33 out of 56 patients, and we identified imaging evidence of spinal cord involvement in 38.3% of these cases (12 out of 33). This rate of spinal cord involvement is notably higher compared to the 5% previously reported by Salvarani et al.^55^. Interestingly, in line with Salvarani et al.'s findings, we also observed that the thoracic cord was the most affected site in cases of spinal cord involvement. Remarkably, spinal cord MRIs revealed abnormalities in two asymptomatic patients who underwent spinal MRI screening. Our findings suggest that spinal cord involvement can occur without accompanying symptoms, and these patients may either remain asymptomatic or develop symptoms related to spinal cord involvement in the future.

In the current study, DSA demonstrated high sensitivity in detecting vasculitis. Within our group, we observed venous phase abnormalities in a pseudophlebitic pattern in 95% of cases. These particular venous phase abnormalities are not visible in CTA or MRA studies and are unique to DSA examination. Panda et al.^56^ reported on clinical and pathological features of three cases of PCNSV in young males, all confirmed through autopsy and without any evidence of systemic disease. One of these cases exhibited granulomatous and other patterns of small vessel vasculitis on histopathological examination, with venulitis, parenchymal hemorrhages, and phlebitis with thrombosis.

In our cohort, we did not observe a high prevalence of arterial abnormalities in the form of stenosis and occlusion as described in cases of PCNSV. Our cohort likely falls into the category of small vessel vasculitis, which is likely to have a normal angiogram compared to medium/large vessel vasculitis^40^. This could be secondary to case selection and lack of DSA in all cases. DSA was done in cases where suspicion of vasculitis was based on MRI findings of micro- and macro hemorrhages and punctate enhancement, but it could not be done in all cases. It may be possible that micro hemorrhage may be more common in small and medium-sized vessel vasculitis cases, and DSA has limitations in detecting all small vessel diseases due to its limited resolution in discerning vessels with a diameter of less than 0.2 mm.

In the present study, brain biopsy biopsies were conducted in 80 cases, with 82.3% (69 patients) being targeted biopsies and 13.7% (11 patients) being non-targeted biopsies. Among these, 56 out of 80 biopsies (70%) revealed positive findings suggestive of PCNSV, while the remaining 24 (30%) were negative. Of the 56 patients with positive biopsy results, 51 had undergone targeted biopsies, and 5 had non-targeted biopsies.

In our investigation, 73.9% (51 out of 69 patients) of targeted biopsies and 45.4% (5 out of 11) of non-targeted biopsies yielded positive results. A study by Miller et al.^57^ revealed that all cases with positive histopathological examinations were associated with targeted biopsies, particularly those that included the leptomeninges. They reported that 26 out of 33 positive biopsies (79%) were confirmatory, while none of the "blind" biopsies (n = 5) yielded positive results (*P* = 0.01). Although Miller et al. did not find any positive biopsies among non-targeted biopsies, our results indicated that 71.4% (5 out of 7 patients) of non-targeted biopsies were also positive. This could be attributed to a diffuse disease process that may not be evident on MRI. Notably, the small sample size of Miller et al.'s study may have contributed to the absence of positive results in blind biopsies. Thus, a targeted meningo-cortical biopsy with a sampling of the leptomeninges should be performed to improve diagnostic accuracy.

In the present group, lymphocytic vasculitis was the most common subtype, similar to the study by Oon et al.^58^ and Sundaram et al.^59^, followed by granulomatous angiitis and necrotizing vasculitis. None of our patients had findings of amyloid angiopathy on histopathology.

PCNSV is a heterogeneous disease, necessitating the recognition that a standardized set of criteria may not universally apply to all its subtypes. In recent times, there has been an effort to redefine the diagnostic criteria for PCNSV, moving away from relying solely on histopathological confirmation. This is because the diagnostic yield of biopsies is often low, as the disease can selectively affect larger and medium-sized vessels, which may not be adequately represented in the biopsy sample. Additionally, although widely used, angiography is not considered the definitive gold standard, as it can overlook the involvement of smaller vessels, and even when positive, it is not 100% specific.

We suggest that certain imaging findings, specifically the presence of hemorrhages (either isolated microhemorrhages or a combination of micro and macrohemorrhages) in SWI, along with dot-linear enhancement, should be incorporated as diagnostic criteria for PCNSV, but only within the context of a comprehensive clinical evaluation. Furthermore, the presence of venulitis detected through DSA should also be considered as a significant marker for PCNSV in the presence of microhemorrhages in SWI.

The strength of the present study is the large number of unselected, consecutive cases with extended follow-up periods, which contributed to a more robust confirmation of the diagnosis while effectively excluding conditions like RCVS or intravascular lymphoma. Compared to individual case reports or smaller previous series, our study cohort stands out as the largest in which SWI has been implemented. Furthermore, we have described a venous abnormality (pseudophlebitis) identified through DSA in PCNSV patients. Additionally, our study encompasses one of the largest PCNSV cases affecting the spinal cord.

The present study is subject to the typical constraint of a retrospective design. The imaging studies were carried out on different scanners with varying strengths and employed diverse protocols, introducing heterogeneity into the data. Additionally, some patients lacked comprehensive laboratory tests and follow-up data. Furthermore, cerebral DSA was not performed for all patients, preventing the demonstration of the pseudophlebitis pattern in all cases. Moreover, among a group of 302 patients who were suspected of having vasculitis based on imaging, only 80 patients underwent a biopsy. Out of those 80 patients, 56 were confirmed to have vasculitis. The remaining patients either declined to have a biopsy or it was not deemed necessary due to the high level of certainty provided by the imaging and clinical presentation. Among the patients who did not undergo biopsy, 20 were diagnosed with secondary vasculitis. None of the patients were found to have infections or systemic lymphomas during the diagnostic process.

Hemorrhages, whether isolated micro or a combination of micro and macrohemorrhages, were identified in SWI, along with dot-linear enhancement, in 96.4% of the patient cohort. In light of these imaging observations, we propose that these findings should be incorporated as a diagnostic criterion for accurately diagnosing PCNSV, provided they are observed in a suitable clinical context and after excluding secondary CNS vasculitis. Moreover, the presence of venulitis on DSA should also be regarded as a potential indicator for PCNSV, particularly when accompanied by microhemorrhages in SWI.

### Supplementary Information

Supplementary Figure 1.

Supplementary Figure 2.

Supplementary Tables.

Supplementary Legends.

## Data Availability

Correspondence and requests for materials should be addressed to A.G. or V.Y.V.

## Abbreviations

AIIMS: All India Institute of Medical Sciences
ANA: Anti-nuclear antibody
ANCA: Antineutrophilic cytoplasmic antibodies
Anti-ds DNA: Anti-double stranded deoxyribonucleic acid
Anti-SSA and SSB: Anti-Sjogren’s syndrome-related antigen A and B
CNS: Central nervous system
CSF: Cerebrospinal fluid
CTA: Computed tomographic angiography
DSA: Digital subtraction angiography
ESR: Erythrocyte sedimentation rate
FLAIR: Fluid-attenuated inversion recovery
GM: Gray matter
HPE: Histopathological examination
IADSA: Intraarterial DSA
ICH: Intracerebral hemorrhage (ICH)
MRA: Magnetic resonance angiography
MRI: Magnetic resonance imaging
mRS: Modified rankin scale
MOG: Myelin oligodendrocyte glycoprotein
NMOSD: Neuromyelitis optica spectrum disorder
PCNSV: Primary central nervous system vasculitis
PVS: Perivascular space
RA: Rheumatoid arthritis
RCVS: Reversible cerebral vasoconstriction syndrome
SAH: Subarachnoid hemorrhage
SWI: Susceptibility-weighted Imaging
T1-WI: T1-weighted imaging
TB-PCR: Tuberculosis- polymerase chain reaction
WM: White matter
VDRL: Venereal disease research laboratory
