# Supplementary figures and images for: The role of susceptibility-weighted imaging & contrast-enhanced MRI in the diagnosis of primary CNS vasculitis: a large case series
Source: Sci Rep. 2024 Feb 27;14:4718. doi: 10.1038/s41598-024-55222-2 (PMC10899183; doi:10.1038/s41598-024-55222-2)

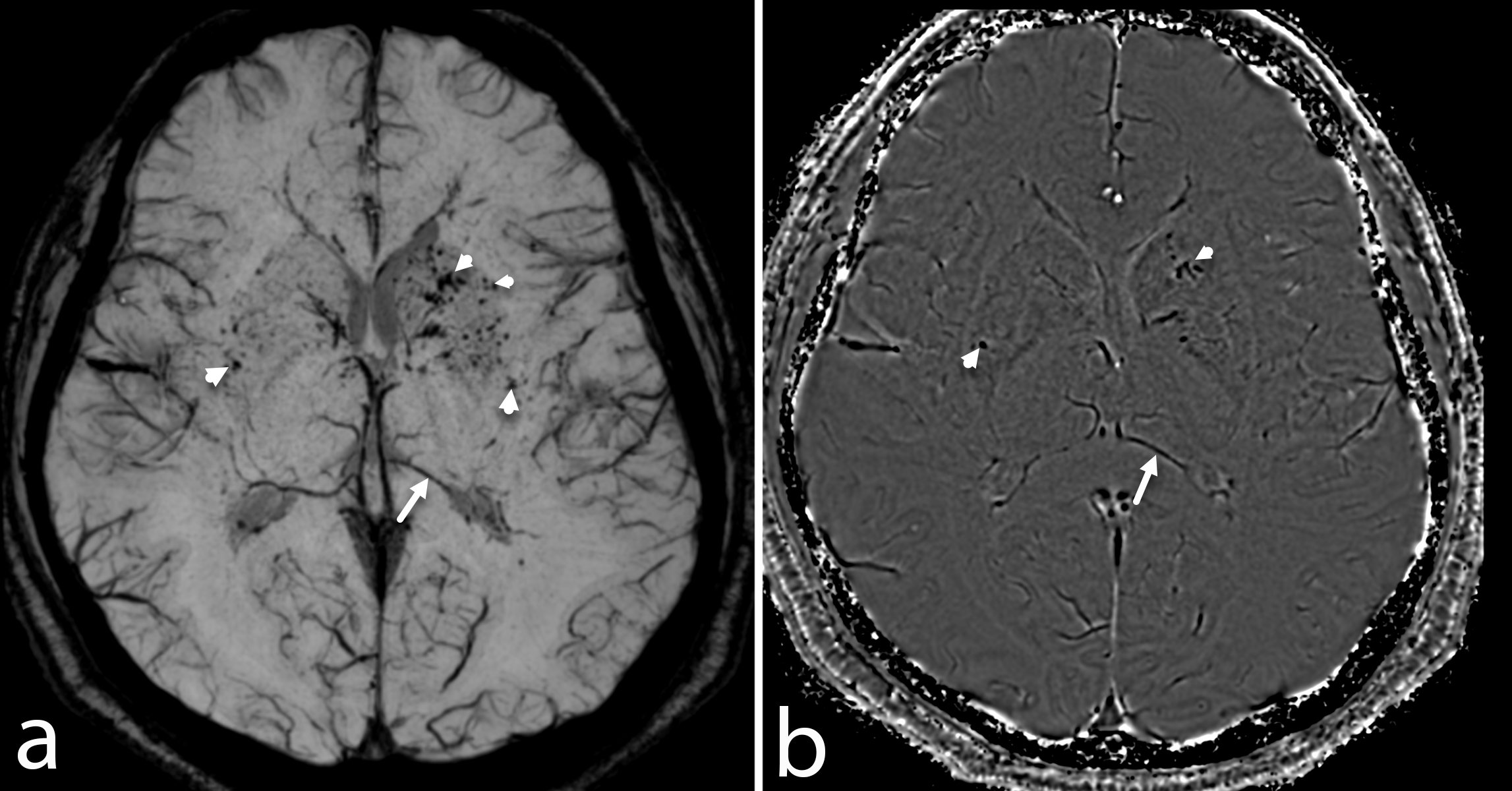

Supplement: Supplementary file 1 — Supplementary Figure 1. [file 41598_2024_55222_MOESM1_ESM.png]

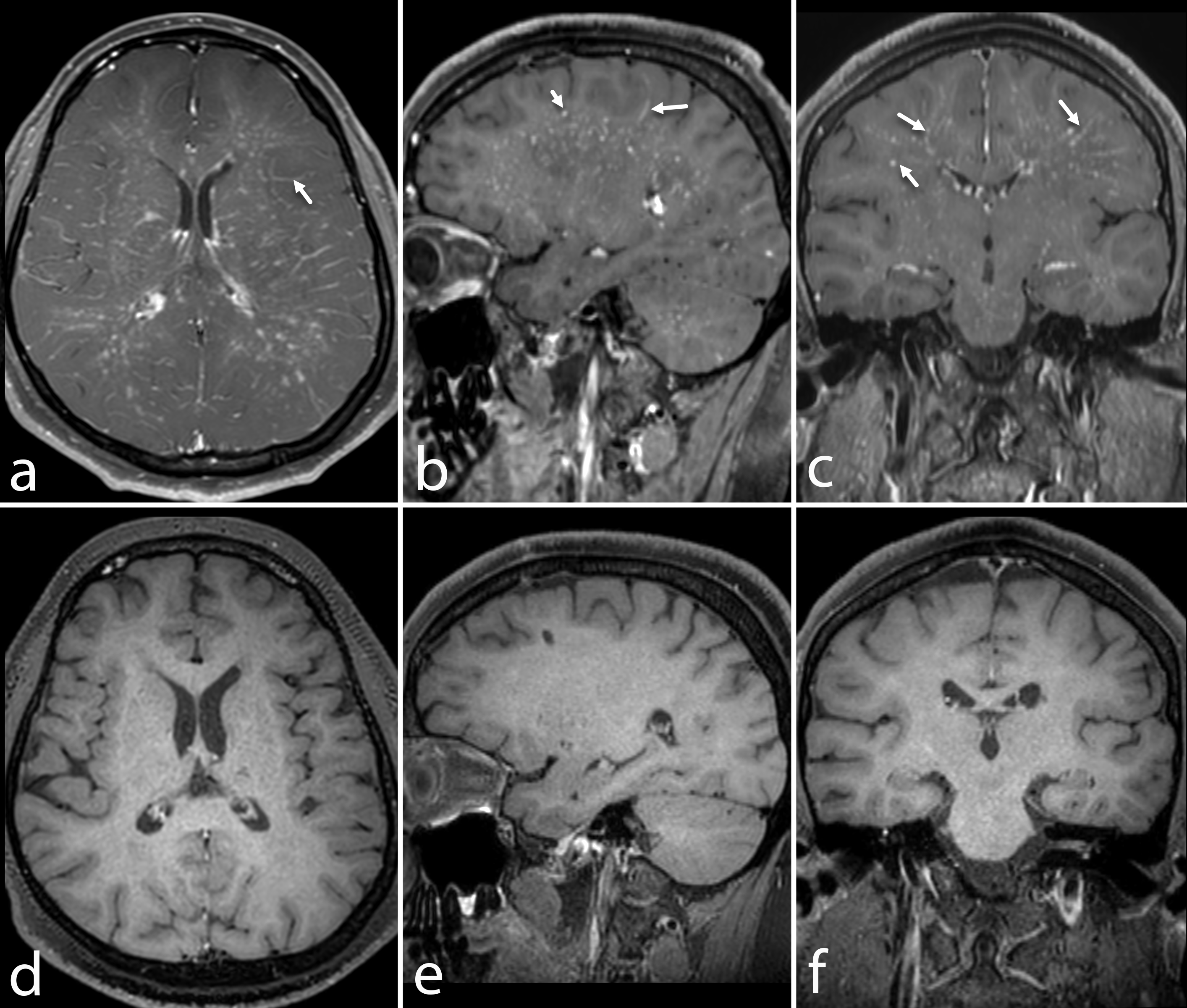

Supplement: Supplementary file 2 — Supplementary Figure 2. [file 41598_2024_55222_MOESM2_ESM.png]
