# Supplementary material for: The role of susceptibility-weighted imaging & contrast-enhanced MRI in the diagnosis of primary CNS vasculitis: a large case series
Source: Sci Rep. 2024 Feb 27;14:4718. doi: 10.1038/s41598-024-55222-2 (PMC10899183; doi:10.1038/s41598-024-55222-2)
Supplement: Supplementary file 3 — Supplementary Tables. [file 41598_2024_55222_MOESM3_ESM.docx]

**Supplementary Table 1: Brain MRI Lesion Distribution**

|  | *T2/FLAIR lesions* | *Hemorrhages* | *Both* | *None* |
| --- | --- | --- | --- | --- |
| **Medulla** | 2 (3.6%) | 7 (12.5%) | 6 (10.7%) | 41 (73.2%) |
| **Pons** | 5 (8.9%) | 9 (16.1%) | 10 (17.9%) | 32 (57.1%) |
| **Midbrain** | 6 (10.7%) | 5 (8.9%) | 6 (10.7%) | 39 (69.6%) |
| **Cerebellum** | 6 (10.7%) | 5 (8.9%) | 28 (50%) | 17 (30.4%) |
| **Frontal Lobe** | 1 (1.8%) | 6 (10.7%) | 46 (82.1%) | 3 (5.4%) |
| **Parietal Lobe** | 1 (1.8%) | 7 (12.5%) | 42 (75%) | 6 (10.7%) |
| **Temporal Lobe** | 4 (7.1%) | 8 (14.3%) | 37 (66.1%) | 7 (12.5%) |
| **Occipital Lobe** | 7 (12.5%) | 3 (5.4%) | 7 (12.5%) | 39 (69.6%) |
| **Caudate** | 9 (16.1%) | 5 (8.9%) | 9 (16.1%) | 33 (58.9%) |
| **Lentiform Nuclei** | 8 (14.3%) | 4 (7.1%) | 18 (32.1%) | 26 (46.4%) |
| **Thalami** | 9 (16.1%) | 10 (17.9%) | 9 (16.1%) | 28 (50%) |
| **Centrum Semiovale** | 1 (1.8%) | 16 (28.6%) | 22 (39.3%) | 17 (30.4%) |
| **Corona Radiata** | 1 (1.8%) | 19 (33.9%) | 26 (46.4%) | 10 (17.9%) |

**Supplementary Table 2:** **Spine MR Imaging Characteristics**

|  | **Definite Vasculitis** |
| --- | --- |
| **Spine MRI done** | 33 (60.0%) |
| At Initial presentation | 21 (63.6%) |
| During follow-up | 12 (36.3%) |
| **Spinal Cord MRI, Abnormal %** | 12/33 (36.4%) |
| Symptomatic | 10/12 (83.3%) |
| Asymptomatic | 2/12 (16.7%) |
| **Spinal cord involvement, %** |  |
| At Initial presentation | 11 (94.1%) |
| During follow-up | 1 (5.9%) |
| **Cord segment** |  |
| Cervical | 3 (25%) |
| Dorsal | 8 (66.7% |
| Conus | 7 (58%) |
| **Lesion extent** |  |
| Short segment | 6 (50%) |
| Long segment | 2 (16.7%) |
| Multifocal | 4 (33.7%) |
| **Spinal Cord expansion** | 7 (58%) |
| **Spinal cord enhancement** | 7 (58%) |
