# Supplementary material for: The role of susceptibility-weighted imaging & contrast-enhanced MRI in the diagnosis of primary CNS vasculitis: a large case series
Source: Sci Rep. 2024 Feb 27;14:4718. doi: 10.1038/s41598-024-55222-2 (PMC10899183; doi:10.1038/s41598-024-55222-2)
Supplement: Supplementary file 4 — Supplementary Legends. [file 41598_2024_55222_MOESM4_ESM.docx]

Legends Supplementary Figures

Figure 1 displays axial SWI images (a) and phase images (b), revealing hyperintensities in the basal ganglia (arrowheads in a & b). These hyperintensities exhibit signal intensities comparable to those of the deep vein (arrow in a & b), indicating that they are likely hemorrhages rather than calcifications.

Figure 2 displays the same patient as shown in figure 1d-f. The patient, a 23-year-old man, experienced sudden vision loss in his left eye. The post-contrast axial (a), sagittal (b), and coronal (c) T1-weighted images reveal a widespread linear perivascular and punctate enhancement pattern in both cerebral and cerebellar hemispheres, as well as the brainstem. After three months of treatment, follow-up images (b-f) demonstrate complete resolution of the enhancement.
